# Mobile-phone-based home exercise training program decreases systemic inflammation in COPD: a pilot study

**DOI:** 10.1186/1471-2466-14-142

**Published:** 2014-08-30

**Authors:** Chun-Hua Wang, Pai-Chien Chou, Wen-Ching Joa, Li-Fei Chen, Te-Fang Sheng, Shu-Chuan Ho, Horng-Chyuan Lin, Chien-Da Huang, Fu-Tsai Chung, Kian Fan Chung, Han-Pin Kuo

**Affiliations:** 1 Department of Thoracic Medicine, Chang Gung Memorial Hospital, 199 Tun-Hwa North Road, Taipei, Taiwan; 2 School of Respiratory Therapy, College of Medicine, Taipei Medical University, Taipei, Taiwan; 3 National Heart & Lung Institute, Imperial College, London, UK

**Keywords:** Chronic obstructive pulmonary disease, Pulmonary rehabilitation, Mobile phone, Biomarker, Interleukin-8

## Abstract

**Background:**

Moderate-intensity exercise training improves skeletal muscle aerobic capacity and increased oxidative enzyme activity, as well as exercise tolerance in COPD patients.

**Methods:**

To investigate whether the home-based exercise training program can reduce inflammatory biomarkers in patients with COPD, twelve patients using mobile phone assistance and 14 with free walk were assessed by incremental shuttle walk test (ISWT), spirometry, strength of limb muscles, and serum C-reactive protein (CRP) and inflammatory cytokines.

**Results:**

Patients in the mobile phone group improved their ISWT walking distance, with decrease in serum CRP after 2 months, and sustained at 6 months. Patients in the control group had no improvement. Serum IL-8 in the mobile phone group was significantly reduced at 2, 3 and 6 months after doing home exercise training compared to baseline. IL-6 and TNF-α were significantly elevated at 3 and 6 months in control group, while there were no changes in mobile phone group. The strength of limb muscles was significantly greater compared to baseline at 3 and 6 months in the mobile phone group.

**Conclusions:**

A mobile-phone-based system can provide an efficient home endurance exercise training program with improved exercise capacity, strength of limb muscles and a decrease in serum CRP and IL-8 in COPD patients. Decreased systemic inflammation may contribute to these clinical benefits. (Clinical trial registration No.: NCT01631019)

## Background

Chronic obstructive pulmonary disease (COPD) is characterized by irreversible airflow obstruction with chronic airway inflammation and emphysematous changes in the lung parenchyma [1,2]. The cardinal symptoms of COPD are dyspnea and/or fatigue, which may result from ventilatory constraints, pulmonary gas exchange abnormalities, peripheral muscle dysfunction, cardiac dysfunction, or combination of the above. COPD is also characterized by persistent chronic inflammation that may extend beyond the pulmonary system, resulting in a state of persistent low grade systemic inflammation [2,3] which has been implicated in various co-morbidities of COPD [4,5]. These extrapulmonary manifestations contribute to morbidity and mortality in COPD patients.

Several systemic inflammatory markers or cytokines including C-reactive protein (CRP), IL-6, IL-8, and tumor necrosis factor (TNF)-α have been associated with COPD risk, COPD mortality, COPD exacerbations, or lung function decline [6-9]. Serum CRP may provide prognostic information about morbidity and mortality in mild to moderate COPD [7,8] with relationships between CRP, IL-6, exercise tolerance, and health status [8,10]. Systemic IL-6 levels are inversely correlated with FEV_1_, and quadriceps strength is independently associated with reduced exercise tolerance [11,12]. IL-8 and TNF-α may be important signaling molecules for neutrophil activation. Increased sputum TNF-α and IL-8 levels have been reported during exacerbations of COPD [13]. Elevated levels of IL-8 have been found in the bronchoalveolar lavage fluid of smokers and COPD patients that correlated positively with neutrophil counts [14]. Circulating levels of IL-8 are elevated during acute COPD exacerbations [15], which contribute to a reduction in health-related quality of life [16]. TNF-α and IL-6 are acute phase proteins known to upregulate the synthesis of CRP, and fibrinogen [17]. The distance walked in the 6-minute walk test was inversely related to serum CRP, IL-6 and IL-8 levels in COPD patients [9]. Taken together, these data suggest that systemic inflammation related to COPD may in part be related to the reduced muscle strength and exercise tolerance observed in COPD patients.

Exercise training is the cornerstone of comprehensive rehabilitation programs in COPD [1]. It improves skeletal muscle oxidative capacity and efficiency with reduced alveolar ventilation at a given work rate [18,19], leading to tolerance of a heavier work load with less dyspnea [20]. Endurance exercise training is clinically efficacious [21]. A recent review supports the notion that mobile technology is a promising way of delivering health services and enhances the exercise training program of pulmonary rehabilitation [1]. Most pulmonary rehabilitation programs are hospital-based and rely on regular supervision and monitoring to achieve persistent and optimal physiological benefits. However, compliance is a major stumbling block [22]. We have developed a home-based exercise training program for stable COPD patients by asking them to walk at a speed controlled by the tempo of music from a program installed on a mobile phone [23]. This system provides an efficient home endurance exercise training program with good compliance and clinical outcomes in patients with moderate-to-severe COPD [23]. We further explored whether the circulating levels of inflammatory markers, such as CRP, IL-6, TNF-α, and IL-8 will be reduced in patients with COPD undergoing a mobile-phone assisted home-based exercise training program [23].

## Methods

### Study subjects

Thirty patients with diagnosis of COPD [with a ratio of forced expiratory volume in one second (FEV_1_) to forced vital capacity (FVC) less than 0.7 after bronchodilators] who had the grading of moderate-to-severe airflow limitation according to GOLD criteria [24] were recruited (Table 1). All subjects were stable within three months prior to enrollment. Exclusion criteria included requirement for oxygen therapy (because these patients were known to be difficult to keep up with mobile-based exercise program) and presence of symptomatic cardiovascular diseases or severe systemic diseases (i.e. hematologic disease, malignancy, systemic lupus erythrosis, end stage renal disease and severe liver cirrhosis) or musculoskeletal conditions with exercise performance limitation. The patients had received regular hospital-based rehabilitation training program once per week, including negative lung expansion therapy, and bicycle riding training, in a clinical stable status at least 3 months. Patients were randomized into either the mobile-phone (n = 14) or the control group (n = 16), and stopped their regular hospital-based rehabilitation program at entry. Two patients of the mobile-phone group and one in control group withdrew because of personal reasons, and one was unwilling to continue after an acute exacerbation. The study was approved by Chang Gung Memorial Hospital Ethics Committee. Written informed consent was obtained from all subjects.

**Table 1:** Characteristics of patients with chronic obstructive pulmonary disease (COPD)

	**Control**	**Mobile phone**	***P- *** **value**
	**(N = 14)**	**(N = 12)**	
Age, yrs	71.9 ± 2.7	71.4 ± 1.9	0.890
Gender, male	14	12	1.000
Body mass index, kg/m^2^	24.5 ± 0.7	22.4 ± 1.0	0.094
SGRQ score	38.1 ± 4.2	32.0 ± 4.0	0.307
Walking distance (ISWT), M	251.4 ± 21.0	261.5 ± 29.9	0.782
FVC, L	1.9 ± 0.1	2.2 ± 0.2	0.158
FVC, % pred.	58.2 ± 4.2	67.5 ± 4.9	0.163
FEV1, L	1.2 ± 0.2	1.5 ± 0.2	0.285
FEV1, % pred.	54.2 ± 6.7	63.5 ± 7.0	0.349
FEV1/FVC, %	63.4 ± 2.9	64.9 ± 4.6	0.784
IC before ISWT, L	1.2 ± 0.1	1.2 ± 0.1	0.731
IC after ISWT, L	1.2 ± 0.1	1.2 ± 0.1	0.615
Delta IC*	−0.04 ± 0.1	−0.05 ± 0.1	0.815

*Definition of abbreviations:* ISWT = incremental shuttle walk test; SGRQ = San George Respiratory questionnaire; FEV1 = forced expiratory volume in 1 s; FVC = forced vital capacity; IC = inspiratory capacity* Change in inspiratory capacity on exercise

Data expressed as mean ± SE.

### Study design

Initially, all subjects were assessed by an incremental shuttle walking test (ISWT) [25] after practice. Patients in the mobile phone group performed daily endurance exercise training under mobile phone guidance, and adherence was reported back to the central server. To ensure subject adherence to the exercise program, at least one health-care member or family learned how to use the exercise program instilled into phone. They opened the mobile phone or helped the patient to answer three questions to confirm successful connection. Then, they got a message from the center server automatically to ensure that the exercise started to be recorded. To exclude the people who may have listened to music but not walked the entire duration, their health-care member or family helped or witnessed the patients to undergo the mobile phone exercise program each time. Endurance walking level was re-assessed and re-adjusted initially on regular clinical visits every four weeks during the first three months. Adherence was reinforced by telephone from health professionals whenever patients missed one day of training. Patients continued their exercise program at home at a fixed walking speed, and returned to the clinic at 1, 2, 3 and 6 months. Adherence to the home-based exercise training program was assessed on the central system every week. Patients in the control group were taught the same exercise protocol, but were verbally encouraged to take walking exercise training at home without any telephone reinforcement. Adherence to the home walking exercise was self-reported. ISWT and venous blood sample for inflammatory biomarkers were performed at baseline, 1, 2, 3 and 6 months.

### Endurance walking exercise with constant intensity

The walking speed of the endurance exercise training at home was set at 80% of maximal capacity predicted from the distance walked during the ISWT [23]. Using the equation of *Predicted VO*_*2*_*peak* (*ml*/*min*/*kg*) = *4.19* + (*0.025* × *ISWT distance*), the peak oxygen uptake (VO_2_peak) of ISWT could be estimated. The level representing 80% of the maximal capacity for endurance walking training at home was derived from 80% of the predicted VO_2_peak value [25]. Patients took another endurance shuttle walk at this calculated level. The number of steps per shuttle was counted. The tempo of music for the appropriate walking speed was calculated based on the walking speed and the number of steps per shuttle according to the equation: *Tempo* (*beats per minute*) = *speed* (*km*/*h*) × *100* × *steps per shuttle* ÷ *60*. Patients then followed this individualized music tempo to walk at a constant speed. The software used for mobile phone monitoring was a Java based application (Java 2 Micro Edition, J2ME) and also provided the music tempo.

### Home-based endurance exercise training

The music software with an individualized preset tempo was installed onto the patients’ mobile phones. Patients turned on the program to walk at a speed following the tempo of music on the mobile phone. Patients turned off the program on the mobile phone when they could not catch up with the tempo. The mobile phone recorded the duration of music played (equal to the duration of walking), and the information was automatically sent to the website server by GPRS for storage.

### Measurements of pulmonary function tests

Forced vital capacity (FVC), forced expiratory volume in one second (FEV1), FEV1/FVC ratio, inspiratory capacity (IC) and breathlessness (rated by Borg’s scale) were measured before and immediately after the ISWT at each hospital visit at 1, 2, 3 and 6 months.

### Measurements of muscle strength

The elbow flexor and knee extensor muscle groups were assessed using a handheld dynamometer microFET2 (Hogan Health Industries, Inc, Draper, UT) [26]. The isometric force of the elbow flexors was assessed with the elbow flexed 90 degrees. The strength of knee extensor muscle was measured with the subject seated with the leg fully extended. Strength was recorded as the peak force of three tests.

### Measurements of CRP, IL-6, IL-8 and TNF-α

Venous blood samples were collected at the same time of day before the exercise training and at baseline, 1, 2, 3 and 6 month. The serum was kept in –70°C freezers. The levels of CRP, IL-6, IL-8 and TNF-α were measured by using highly sensitive enzyme linked immunosorbent assay kits (Alpha Diagnostics, San Antonio, TX, USA). The median interassay coefficient of variation was 6.3%.

### Statistical analysis

Data were presented as mean ± SE. The baseline data of the mobile phone and the control group were evaluated by descriptive statistics and analyzed by Student's test. The data of the mobile phone group and the control group or between the two groups from baseline to 6 months were determined by analysis of variance with repeated measures. A *p* value < 0.05 was considered significant.

## Results

The two groups were comparable in terms of the severity of COPD (Table 1). The subjects in mobile-phone group performed walking exercise at home at a frequency of around 4 to 6 days per week throughout the 6 months. Only 50% of patients (n = 7) in control group reported by themselves that they still kept regular walking at the end of study.

### Walking distance under ISWT

**Figure 1:**
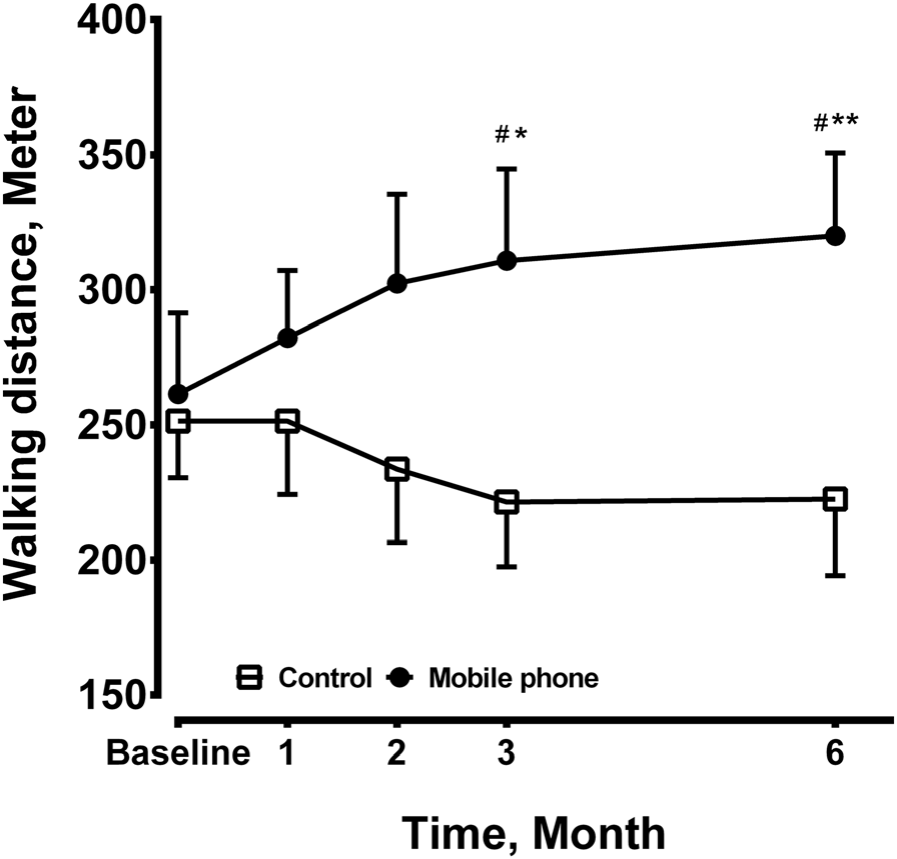
**Incremental shuttle walking test (ISWT) was performed in the mobile phone group (N = 12) and the control group (N = 14) at baseline and after 1, 2, 3, 6 months.** The sample size was 12 in the mobile phone group and 14 in the control group at each time point. Results are shown as mean ± SE. *P < 0.05, **P < 0.01 compared to the baseline. #P < 0.05 compared to the control group.

In the mobile phone group, there was an improvement in walking distance at 3 months (310.8 ± 34.0 M, n = 12, p < 0.05), and at 6 months (320.0 ± 30.7 M, n = 12, p < 0.01) compared to the baseline (261.5 ± 29.9 M, n = 12, analysis of variance with repeated measures) (Figure 1). However, in the control group, walking distance (251.4 ± 21.0 M, n = 14, baseline) was going down over time (222.5 ± 28.3 M, n = 14, at 6 months), but no significance was achieved (Figure 1). The walking distance of the mobile phone group was significantly increased compared to the control group at 3 and 6 months.

### Muscle strength

The strength of the 4 tested upper and lower extremity muscle groups were the same at baseline in both groups (Table 2). The muscle strength of both upper extremities increased in the mobile phone group (p < 0.01) at the second, third and sixth months compared with baseline. After 3 and 6 months of mobile phone-based home walk training, the strength of lower limb muscles was significantly greater compared to baseline. The strength of lower limb muscle in the mobile phone group was significantly greater than that of the control group. In contrast, the strength of upper or lower limb muscle in the control group did not show any change throughout the study period compared with baseline.

**Table 2:** Evaluation of limb muscle strength

	**Baseline**	**1 M**	**2 M**	**3 M**	**6 M**	^**a**^**p**
**Control group**	**n = 14**	**n = 14**	**n = 14**	**n = 14**	**n = 14**	
**Elbow flexion, kg**						
**Left**	13.3 ± 0.7	12.2 ± 0.7	12.6 ± 0.5	13.0 ± 0.6	13.1 ± 0.5	0.354
**Right**	13.6 ± 0.8	12.4 ± 2.9	13.0 ± 0.5	13.3 ± 0.7	13.2 ± 0.6	0.953
**Knee extension, kg**						
**Left**	12.2 ± 0.9	10.5 ± 0.7	12.6 ± 0.8	12.8 ± 0.6	12.8 ± 0.6	0.098
**Right**	12.0 ± 0.8	10.5 ± 0.7	12.2 ± 0.7	12.7 ± 0.6	12.7 ± 0.6	0.114
**Mobile phone group**	**n = 12**	**n = 12**	**n = 12**	**n = 12**	**n = 12**	
**Elbow flexion, kg**						
**Left**	11.8 ± 0.5	12.4 ± 0.6	12.9 ± 0.5*	13.3 ± 0.5**	13.5 ± 0.5**	0.015
**Right**	11.5 ± 0.6	12.6 ± 0.5	13.5 ± 0.4*	14.3 ± 0.5***	14.7 ± 0.4***	<0.001
**Knee extension, kg**						
**Left**	10.9 ± 0.8	11.8 ± 0.8	12.3 ± 0.7	13.7 ± 0.9	14.7 ± 0.7*^#^	0.016
**Right**	10.8 ± 0.8	11.5 ± 0.7	12.0 ± 0.8	13.9 ± 0.9*	15.1 ± 0.7**^#^	0.002

*Definition of abbreviations*: M, month; kg, kilogram. Data present as mean ± SE.

^a^P value was determined by analysis of variance with repeated measures.

*P < 0.05, **P < 0.01, ***P < 0.001 compared to the baseline of corresponding group. ^#^p < 0.05 compared to the control group at the same time.

### Serum CRP, IL-6, IL-8 and TNF-α

**Figure 2:**
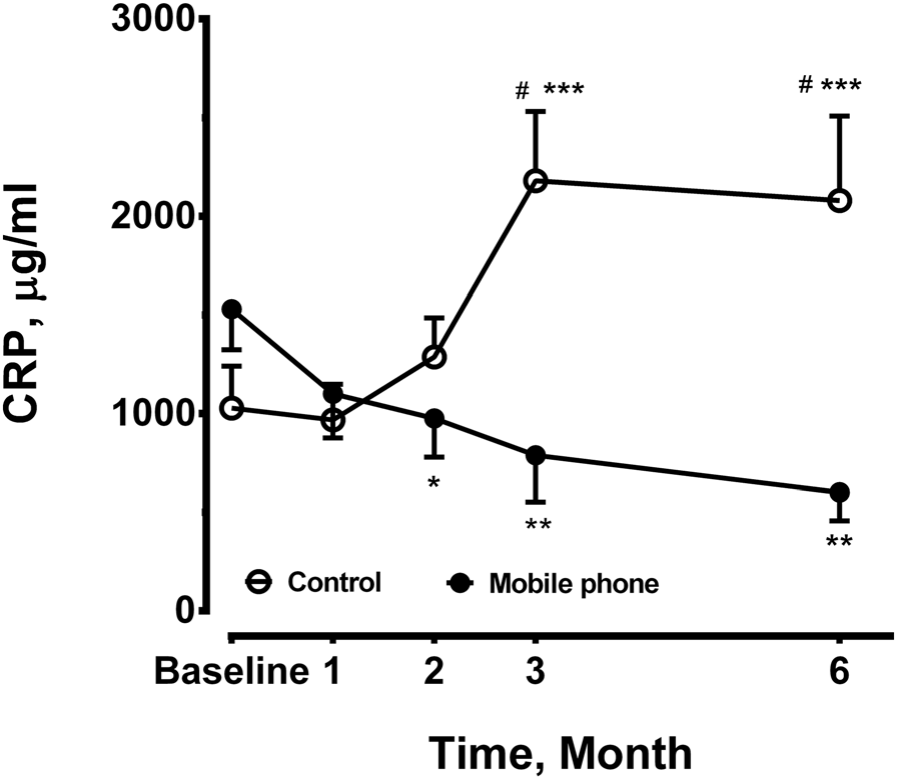
**Plasma levels of C-reactive protein (CRP) were measured in the mobile phone group and control group at baseline, 1, 2, 3 and 6 months.** The sample size was 12 in the mobile phone group and 14 in the control group at each time point. *P < 0.05, **P < 0.01, ***P < 0.001compared with the baseline level of corresponding group. #P < 0.05 compared to the control group.

Plasma levels of CRP in mobile phone group were decreased after 2 months (975.3 ± 197.3 μg/ml, n = 12, p < 0.05), 3 months (788.3 ± 237.0 μg/ml, n = 12, p < 0.01) throughout 6 months (601.1 ± 144.5 μg/ml, n = 12, p < 0.01) compared to baseline (1531.0 ± 206.4 μg/ml, n = 12, analysis of variance with repeated measures). Levels of CRP were increased at 3 months (2181.0 ± 352.5 μg/ml, n = 14) and 6 months (2080.0 ± 428.4 μg/ml, n = 14) compared to baseline (1028.0 ± 213.1 μg/ml, n = 14, p < 0.001, analysis of variance with repeated measures) in the control group (Figure 2). Plasma levels of CRP in the control group were significantly increased compared to the mobile phone.

**Figure 3:**
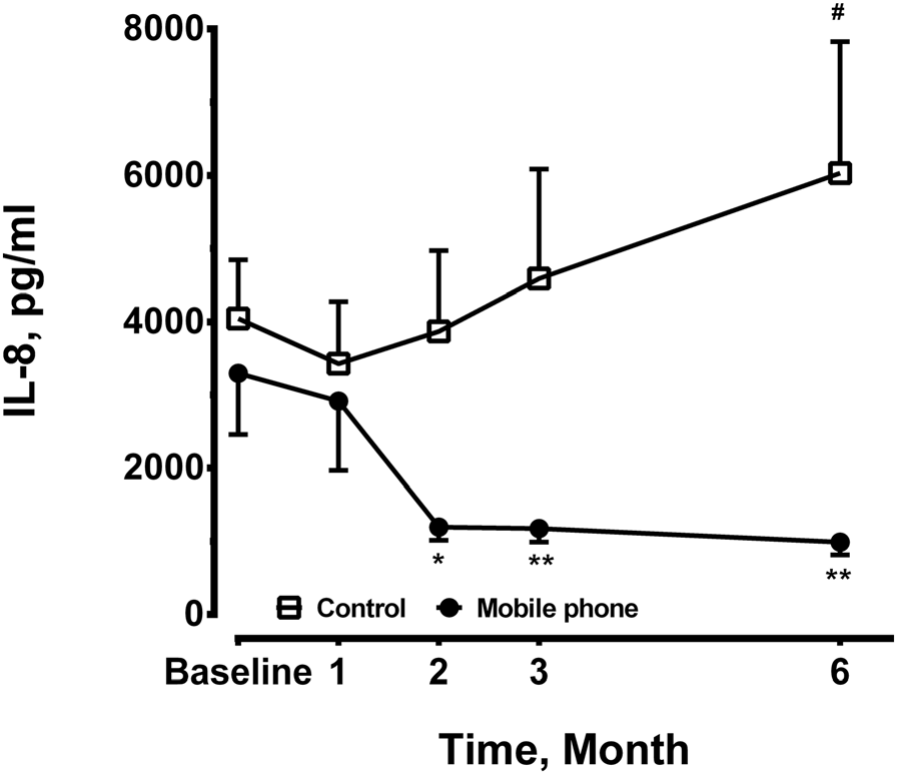
**Plasma levels of interleukin (IL)-8 were measured at baseline, 1, 2, 3 and 6 months in the mobile phone group (n = 12) and the control group (n = 14)**. *P < 0.05, **P < 0.01 compared to the baseline level of the mobile phone. #P < 0.01 compared to the control group at the same time point.

Plasma levels of IL-8 in mobile phone group was significantly reduced at 2 months (1196.0 ± 183.1 pg/ml, n = 12, p < 0.05), 3 months (1179.0 ± 186.2 pg/ml, n = 12, p < 0.01), through 6 months (990.1 ± 175.6 pg/ml, n = 12, p < 0.01) on home exercise training compared to the baseline (3299.0 ± 839.4 pg/ml, n = 12). The circulating levels of IL-8 did not change (Figure 3). In the control group at 6 months, plasma levels of IL-8 in the control group were higher compared to the mobile group.

**Figure 4:**
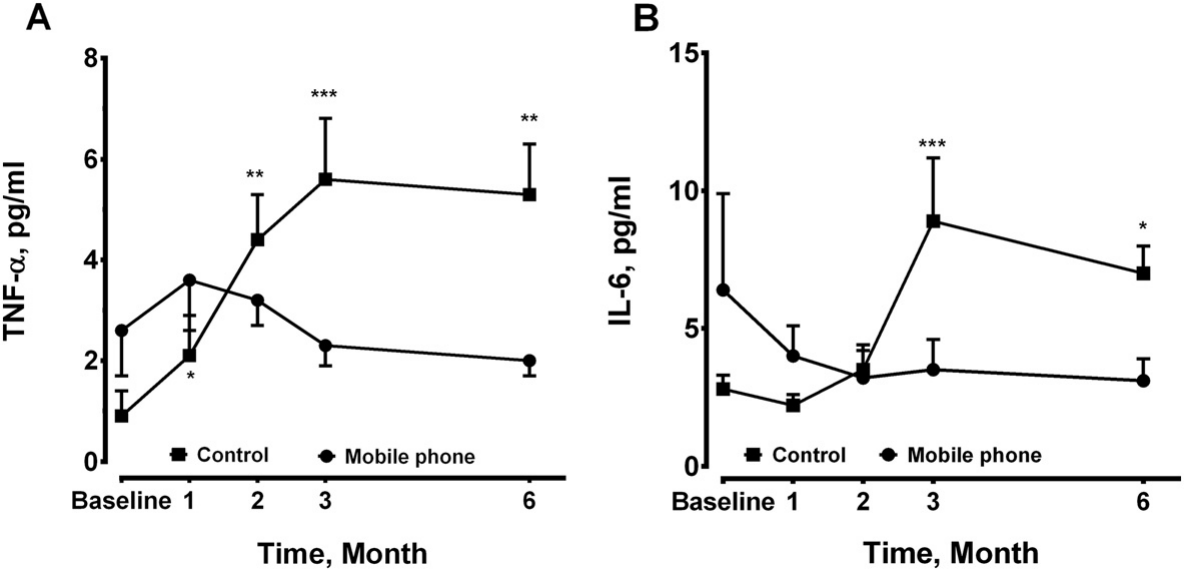
**Plasma levels of (A) tumor necrosis factor (TNF)-α and (B) interleukin (IL)-6 were measured at baseline, 1, 2, 3, 6 months in both groups.** The sample size was 12 in the mobile phone group and 14 in the control group at each time point. *P < 0.05, **P < 0.01 ***p < 0.001 compared to the baseline levels of the control group.

Levels of TNF-α did not show any difference during the period of home exercise training program in the mobile phone group (Figure 4A). By contrast, they were significantly elevated at 2, 3 and 6 months in the control group (Figure 4A). Serum levels of IL-6 was increased at 3 months (8.9 ± 2.3 pg/ml, n = 14, p < 0.001) and 6 months (7.0 ± 1.0 pg/ml, n = 14, p < 0.05) compared to the baseline (2.8 ± 0.5 pg/ml, n = 14), while levels of IL-6 in the mobile phone group remained unchanged (Figure 4B).

## Discussion

We demonstrated that COPD patients, under the assistance of a mobile phone, were able to perform a home-based, individualized endurance exercise training program at an intended walking speed controlled by a pre-set tempo of music. During the six month follow-up period of home-based exercise training, exercise capacity, strength of limb muscles and inflammatory biomarkers of CRP and IL-8 were improved in the group under mobile phone guidance, but not in control group. In control group, who did not benefit from regular home-exercise training in the COPD patients, the level of proinflammatory cytokines of IL-6 and TNF-α remained elevated at 3 and 6 months. Our work indicates for the first time that long-term home exercise training using an individualized design and adjusted training protocol can reduce the inflammatory biomarkers, CRP and IL-8, which is likely the result of an improvement in systemic inflammation.

CRP is an acute-phase reactant that can be upregulated sensitively during systemic inflammation, and is associated with muscle mass, strength, physical function, and disability [27]. CRP levels can be reduced in healthy subjects receiving 9 months of endurance exercise training [28]. Increased concentrations of CRP and IL-6 were associated with impaired physical function in older adults with COPD [29]. In the control group, there was a gradual increase in levels of proinflammatory cytokines, IL-6, TNF-α and CRP in patients who performed home exercise training of free walk. COPD patients are likely to become de-conditioned gradually due to dyspnea and systemic inflammation [30], and may therefore show significant cytokine response at low levels of activity. Regular exercise may induce anti-inflammatory effects [31]. Our previous study showed that under the mobile-phone assisted home exercise training program has improved exercise capacity, breathlessness, quality of life, inspiratory capacity and air-trapping in patients with COPD [23]. In this study, we extend further the benefits of this program by showing that the strength of upper and lower limb muscles in COPD patients increased after 2 or 3 month of regular exercise. Thus, a mobile phone-based home exercise training program can provide an effective training for COPD patients. Long-term exercise training can improve systemic inflammatory biomarkers in patients with COPD.

The presence of systemic inflammation has been suggested by studies [32,33] showing systemic oxidative stress, the activation of circulating neutrophils and lymphocytes, and increased plasma levels of TNF-α. The presence of oxidative stress in the airways of smokers and patients with COPD has been shown by increased products of lipid peroxidation and altered antioxidant status [34]. Transcription factors such as nuclear factor-κB (NF-κB) and activator protein-1 (AP-1), which are oxidant-sensitive, are important for gene transcription of the inflammatory cytokines, and associated with upregulated airway inflammation in COPD. Proinflammatory cytokines, such as IL-8, TNF-α and IL-6 [34,35] are oxidant-sensitive proteins. For example, IL-8 gene has both NF-kB and AP-1 binding sites in the promoter region that regulates its transcriptional activation [36]. Exercise training improves skeletal muscle oxidative capacity and efficiency that leads to less alveolar ventilation for a given work rate [18,19]. Patients can tolerate a heavier work load with less dyspnea on exercise [37]. The lower limb muscles play an important role in exercise training programs because the quadriceps muscles are the major muscles for patients’ mobilization [38]. In our study, the upper limb muscles also benefit from the paced-walk exercise training program, possibly related to the coordinated training high-intensity exercise with swing and balance of upper limbs.

Our results demonstrated that the serum levels of TNF-α and IL-6 were increased in the control group. The effects of peripheral muscle training on specific systemic inflammatory mediator levels (TNF-α, IL-6 and CRP) have been proposed to be associated with muscle dysfunction in COPD [39,40]. Endurance exercise training in COPD can induce peripheral muscle adaptation and modification by factors regulating skeletal muscle hypertrophy and regeneration [41], but in the absence of a decrease in systemic or local muscle TNF-α and IL-6, similar to our results in the mobile phone group. In addition, people with COPD who walked the most had the lowest plasma CRP and IL-6 levels [42]. The systemic inflammatory process, evaluated by serum IL-6 and TNF-α which may result from pulmonary inflammation and oxidative stress or hypoxia [40,43], seems to be persistent and progressive in COPD patients [42,44]. In our study, COPD patients in the control group may have less intensity of endurance walking training after stopping regular hospital-based pulmonary rehabilitation. They exhibited s raised plasma levels of TNF-α and IL-6 at 3 and 6 months compared to those in the mobile phone group. TNF-α and IL-6 are acute phase proteins known to upregulate the synthesis of CRP and fibrinogen [17]. TNF-α activates the transcription factor, nuclear factor-κB (NF-κB), that switches on transcription of the IL-8 gene and increases IL-8 release from the airway epithelium and neutrophils [36]. Our results also showed a significant increase in serum levels of CRP (at 3 and 6 months) and IL-8 (at 6 months) compared to the mobile phone group. These results provide the conceptual basis that walking leads to a reduction in systemic inflammation in COPD patients.

In the control group, one would expect that the walking distance may not be going up at the same rate as the intervention group, but would hopefully at least remain static than decline at 6 months. This finding had been reported in the control group of Wijkstra et al [45]. One possibility is that all subjects in our control group received regular hospital-based rehabilitation of bicycling training once per week, which can successfully maintain functional exercise capacity [46]. Without a supervised intervention, subjects of control group may decrease the time or intensity of home walking program. Therefore, their incremental shuttle walk distance only declined gradually. 6MWT is a self-paced walking test of fixed distance, while ISWT is a paced test of an indefinite duration. The presence of quadriceps muscle fatigue was more frequent and more pronounced after cycling than after walking. Another possibility is that the incremental shuttle walk in our study is more responsive to the effects of pulmonary rehabilitation than 6 minute walk test (6MWT) [47]. In another study [48], the walking distance appear to decline by twelve months after stopping pulmonary rehabilitation program.

Exercise training is the cornerstone of pulmonary rehabilitation [1,11]. High intensity training significantly improved patients’ exercise capacity and physiologic adaptation during endurance training [49]. COPD patients presented with increased pulmonary and systemic oxidative stress after simple exercise compared with healthy control subjects [50]. Unplanned exhausted exercise in COPD patients may lead to fatigue and contribute to exercise limitation related to the excessive load placed on inspiratory muscles, and deteriorate systemic inflammation [51]. Through well-designed evaluation of patients’ exercise capability, our mobile phone based program provides a real-time monitoring about patients’ daily exercise training, and dyspnea sensation. The central monitoring of patients’ physical activity offers the opportunity for early intervention and management in COPD patients to prevent exacerbation of disease. This specific computer-based monitoring strategy may further reduce the medical costs through the planned exercise training which reduced systemic inflammation, and improved patients’ adherence through daily communication.

A randomized clinical trial showed that weekly telephone contacts and monthly supervised rehabilitation sessions produced only modest effects in maintaining improvements in exercise tolerance and ratings of overall health status [52]. The exercise tolerance declined gradually in the usual care group. In contrast, distance walked on the endurance shuttle waking test (ESWT) significantly improved either in the hospital or community rehabilitation groups. There was no difference in exercise tolerance between hospital or community groups with the telephone and no-telephone groups [53]. Our work showed a greater improvement of exercise in COPD patients by mobile phone home-based exercise program. Effectiveness of intervention or contact or combined with an optimal method, such as cell phone, for incorporating rehabilitation strategies may have been critical in motivating participants to continue exercise.

The present study indicates that COPD patients can substantially benefit from rehabilitation with lower systemic inflammation, even though hospital based exercise training once per week. Several home-based rehabilitation programs have been developed with proven clinical benefits in quality of life and exercise tolerance [54,55]. In those programs for which walking freely for a period of time is recommended, it is more difficult to establish the walking speed necessary to achieve a training effect at home [56]. The long-term adherence to exercise training is the critical factor in sustaining the clinical benefit in the home setting program. It declines when regular monitoring or supervision is removed [57], like our control group with progressive elevation of cytokines. The current cell phone-assisted system may provide an efficient home-based exercise training program, due to its controlled exercise workload by pacing walking and web-based supervision of exercise performance and low-grade systemic inflammation.

The limitations of this study are its relatively small patient numbers, and short-term follow-up period. However, these results demonstrate that intensive daily exercise programs without undue exhaustion can be performed successfully on an out-patient basis through a mobile phone based communication. This tele-rehabilitation program combined the advantage of both the self-management strategies and the direct investigation from the health care groups via computer monitoring. Further longitudinal observation for outcomes which includes decline in pulmonary function, deterioration in muscle endurance, as well as the systemic inflammatory mediators may lead to useful measures to overcome detrimental changes in COPD patients.

## Conclusions

Mobile-phone-based system provides a feasible, efficient home paced-walk endurance exercise training program with good compliance and clinical outcomes in improving exercise capacity, strength of limb muscles and reducing inflammatory biomarkers, such as CRP and IL-8, in patients with COPD. The improved systemic inflammation may contribute to clinical benefits such as a reduction in acute exacerbation. Exercise training tailored to address the individual patient factor to optimal exercise loading and intensity via surveillance through mobile phone communication could play an important role in optimising long-term outcomes and prevent functional derangement in COPD patients.

## Abbreviations

COPD: Chronic obstructive pulmonary disease; ISWT: Incremental shuttle walk test; CRP: C-reactive protein; IL-8: Interleukin-8; GPRS: General packer radio service; VO2peak: Peak oxygen uptake; FVC: Forced vital capacity; FEV1: Forced expiratory volume in one second; IC: Inspiratory capacity; BMI: Body mass index.

## Competing interests

The authors declare that they have no competing interests.

## Authors' contributions

CHW performed the work of part of design, acquisition of data and drafting the article. PCC contributed to the acquisition of data, as well as part work of writing article. WCJ, LFC, TFS, and SCH contributed to the data collection and education of exercise training program for patients. HCL, CDH, and FTC worked on the acquisition and analysis of data. KFC contributed to study concept and design, interpretation of data, and revising the manuscript critically for important intellectual content. HPK took the substantial responsibility to main concept and design of this work and contributed to the analysis and interpretation of data and final approval of the final version. All authors read and approved the final manuscript.

## Pre-publication history

The pre-publication history for this paper can be accessed here:

http://www.biomedcentral.com/1471-2466/14/142/prepub
